# From 30 million to zero malaria cases in China: lessons learned for China–Africa collaboration in malaria elimination

**DOI:** 10.1186/s40249-021-00839-y

**Published:** 2021-04-20

**Authors:** Jun-Hu Chen, Jun Fen, Xiao-Nong Zhou

**Affiliations:** 1 NHC Key Laboratory of Parasite and Vector Biology, National Institute of Parasitic Diseases, Chinese Center for Disease Control and Prevention, Chinese Center for Tropical Diseases Research, WHO Collaborating Centre for Tropical Diseases, National Center for International Research on Tropical Diseases, Ministry of Science and Technology, Shanghai, 200025 People’s Republic of China; 2 The School of Global Health, Chinese Center for Tropical Diseases Research, Shanghai JiaoTong University School of Medicine, Shanghai, 200011 People’s Republic of China

**Keywords:** Malaria, Elimination, China, Africa, Genetic epidemiological methods

## Abstract

Malaria was once one of the most serious public health problems in China, with more than 30 million malaria cases annually before 1949. However, the disease burden has sharply declined and the epidemic areas has shrunken after the implementation of an integrated malaria control and elimination strategy, especially since 2000. Till now, China has successfully scaled up its efforts to become malaria-free and is currently being evaluated for malaria-free certification by the WHO. In the battle against malaria, China’s efforts have spanned generations, reducing from an incidence high of 122.9/10 000 (6.97 million cases) in 1954 to 0.06/10 000 (7855 cases) in 2010. In 2017, for the first time, China reached zero indigenous case of malaria, putting the country on track to record three consecutive years of zero transmission by 2020, accoding to the National Malaria Elimination Action Plan (2010–2020). China’s efforts to eliminate malaria is impressive, and the country is dedicated to sharing its lessons learned in malaria elimination—including, but not limited to, the application of novel genetics-based approaches—with other nations through new initiatives. China will promote international relationships and establish collaborative platforms on a wide range of topics in roughly 65 countries, including 20 African nations. China’s experience in applying innovative genetics-based approaches and tools to characterize malaria parasite populations, including surveillance of markers related to drug resistance, categorization of cases as indigenous or imported, and objective identification of the likely sources of infections to inform efforts towards malaria control and elimination in Africa could offer game-changing results when applied to settings with ongoing transmission.

## Background

Malaria has been documented in traditional Chinese medicine books, and the presence of malaria goes back approximately 4000 years in China’s history. Malaria expanded broadly, especially in rural regions, and outbreaks occurred often over the last few decades, with an estimated 90% of the population at risk of infection in the 1940s (i.e., 30 million cases and 300 000 deaths reported annually) [1]. In 1955, the National Malaria Control Programme was established to engage communities to confront the burden of malaria. Although the disease remained highly endemic, with large-scale outbreaks in the 1960s–1970s, steady progress towards malaria control was made (only 117 000 cases were reported in 1990). Nevertheless, after the implementation of an integrated strategy for malaria control, including interventions, as well as socio-economic and environmental development, such as urbanization, alterations in the natural surroundings which affected the transmission pattern including changes of malaria vectors’ distribution, the occurrence of indigenous malaria cases has been steeply reduced, and epidemic regions have been drastically shrunken [2, 3]. Thus in 2010, as part of the United Nations’ 2000 Millennium Development Goals, China’s National Health and Family Planning Commission recruited cooperation from 13 ministries to support the ambitious for National Malaria Elimination Action Plan (2010–2020), which aimed to halt and reverse the incidence of disease by 2015 and eliminate local transmission by 2020 [4]. The country has effectively beaten back malaria within its borders, with no indigenous cases reported since 2017—the first such report in the country’s history [5]. The country will soon receive global recognition for achieving malaria-free status.

This astounding progress—by the most populous country in the world—has been supported by multiple innovations in malaria treatment and surveillance. Anticipating the need for novel anti-malarial compounds in the 1960s, the national government collaborated with the China Academy of Traditional Chinese Medicine to screen potential natural products based on traditional Chinese medicine. These efforts culminated in the identification of the anti-malarial properties of the active product of* Artemisia*, artemisinin, in 1971 by Professor Tu You You. In 2015, she became the first Chinese winner of the Nobel Prize in Physiology or Medicine [6]. Today, artemisinin remains the world’s primary lifesaving anti-malaria drug used around the world. China’s innovative surveillance methods, particularly the “1-3-7” approach—wherein the National Malaria Elimination Programme disseminates and monitors the tenets of the national elimination strategy by describing a key set of targets, responsibilities, actions, and time frames—has served as a model for malaria control in other countries [7]. The approach has been widely lauded, including by the World Health Organization. China’s efforts to partner with malaria-endemic countries to combat the disease has enabled meaningful connections in terms of providing guidance and support to control and eliminate malaria in Africa and other parts of the world.

In order to further distill Chinese lessons learnt from the National Malaria Elimiation Programme and to learn about China’s journey—“From 30 Million to Zero Malaria Cases in China: Lessons Learned for Malaria-Eliminating Countries in Africa”, the National Institute of Parasitic Diseases at China CDC in collaboration with Harvard T.H. Chan School of Public Health and the World Health Organization’s Global Malaria Programme to convene a special two-day’s online symposium to delve into the five specific topics in greater detail on December 7–8, 2020. The five topics discussed during the workshop was (i) to share with the malaria control and elimination experience in China; (ii) to understand the important role of strengthening the construction of the public health system, scientific and technological research in eliminating malaria and consolidating achievements; (iii) progress in global malaria elimination and challenges facing the COVID-19; (iv) to apply genetic methods to the technical support of eliminating malaria and improving public health; (v) to strengthen cooperation with African countries in malaria control and research. Main outcomes and conclusions of the symposium are presented in this paper.

## China’s leadership in global malaria eradication efforts

China was once a major malaria-endemic country, but the Chinese government attaches great importance to malaria prevention and control. After 70 years of hard work, there have been no reports of indegenous cases for nearly 4 consecutive years since 2017, reaching the World Health Organization's elimination standard. In November 2020, it has formally applied to the World Health Organization for national malaria elimination certification.

Scientific research institutions at all levels in China have carried out a series of laboratory and field studies on malaria control and elimination technology, which have been applied in the process of malaria control and elimination in China, providing scientific basis and technical support for the smooth control of malaria epidemic and the realization of malaria elimination goals in China. In the long-term battle with malaria, China has discovered and extracted artemisinin, a special anti-malarial drug, from hundreds of Chinese herbal medicines, which has made significant contributions to the prevention, control and elimination of malaria in the world. The national malaria network reporting system and laboratory testing network have been established [8], the malaria vector resistance and *Plasmodium* species resistance monitoring system have been improved, the work strategy of "tracking clues, counting and pulling out the source" has been formulated [9], and the case report, investigation and foci disposal for the "1-3-7" work model and the "3 + 1 line of defense" in border areas have also been developed [10]. Among them, the “1-3-7" work model is the global work model for malaria elimination, and it is officially written into the technical documents of the World Health Organization for global promotion and application.

## A malaria-free China invests in academic partnerships and establishes a China-African collaboration network

Over the past decade, China's multi-billion-dollar investment in health-care reform has enabled the country to make incredible progress toward key public health challenges—with a focus on programmes aimed at saving the lives of mothers and children and improving management and development of public health. Today, China continues to innovate in its path to malaria elimination by seeking to incorporate genetic tools to inform its efforts and is currently applying technological advances in genetic epidemiology [11, 12].

Although the global new crown epidemic is still spreading and the risk of spreading is increasing, all countries and people uphold the concept of a community with a shared future for mankind, and help each other to fight the epidemic. With the support of the international community, the Chinese government and people through arduous efforts have created another heroic feat in the history of mankind’s struggle against disease. China–Africa friendship is getting better with each passing day, and China–Africa cooperation in the field of health has a long history. Since China sent its first foreign medical team to Algeria in 1963, medical aid to Africa has never been interrupted. In December 2015, President Xi Jinping proposed the “China–Africa Public Health Cooperation Plan” at the Johannesburg Summit of the Forum on China–Africa Cooperation. In September 2018, at the Beijing Summit of the 7th Forum on China–Africa Cooperation, health was listed as building the destiny of China and Africa. One of the “eight major actions” implemented by the community, in June 2020, at the China–Africa Solidarity Anti-epidemic Summit, it was emphasized to jointly build a China–Africa health community. China hopes to strengthen malaria cooperation with African countries and further promote it as an important part of China–Africa health cooperation. China will work together with the international community to jointly fight the new crown pneumonia epidemic and promote the global elimination of malaria, and jointly build a global community with a shared future for mankind.

## Application of genetic methods to the technical support of eliminating malaria and improving public health

China is exploring genetic technology to assist smart decision-making, and applying new genetic epidemiological methods to promote the surveillance of malaria elimination. With the support of the Harvard Global Research Fund, the National Institute of Parasitic Diseases, Chinese Center for Disease Control and Prevention has established an academic partnership with Harvard University, Harvard T. H. Chan School of Public Health, and Broad Institute of MIT and Harvard. Committed to sharing experiences in the application of new genetic technology with countries through cooperation mechanisms such as the "Belt and Road Initiative". In the future, China will establish a broader cooperation platform with the "Belt and Road" countries including African countries to support and promote global malaria prevention and control work in the future.

With support from the Harvard Global Institute, an academic partnership to advance data-driven decision-making methods was formed among researchers from the National Institute of Parasitic Diseases, Chinese Center for Disease Control and Prevention, faculty from Harvard University and the Harvard T. H. Chan School of Public Health, and scientists at the Broad Institute of MIT and Harvard. By employing DNA sequencing and advanced genomic-based tools to understand and predict parasite movement and to differentiate imported from indigenous parasites, this scientific partnership offers a road map for other countries seeking to control the disease across different elimination settings in Africa.

China’s efforts to partner with malaria-endemic countries to combat the disease has enabled meaningful connections in terms of providing guidance and support to control and eliminate malaria in Africa and other parts of the world.

## Panel: strengthen cooperation with African countries in malaria control and research

A set of control strategies and measures for National Malaria Elimiantion Action Plan in different local settings has been implemented successfully over the last 60 years in China. This experience has included collaboration between China and Africa in the field of malaria control, in the larger context of China’s contribution to development in Africa as a major trading and investment partner. In early 2013, the government of China has promised to continue increasing its investment in Africa, which includes medical assistance as well as promotion of sustainable development. The National Health Commission and the Ministry of Commerce of China have proposed to provide technical support for malaria control and elimination in Africa, which would further promote South–South cooperation through mutual exchanges in the field of malaria control and elimination. The cooperation between China and Africa will not only strengthen China’s capacity in engagement in global health, but will also provide a platform to share health products (e.g. quality control in production and delivery of antimalarial drugs), techniques (e.g. diagnostics) and intervention strategies. Based on multilateral communication, P.R. China intends to develop malaria prevention and control projects in several African countries together with international experts working in Africa.

## Collaborative research scopes

China’s experience in applying innovative genetics-based approaches and tools to characterize malaria parasite populations, including surveillance of markers related to drug resistance, categorization of cases as indigenous or imported, and objective identification of the likely sources of infections to inform efforts towards malaria control and elimination in Africa could offer game-changing results when applied to settings with ongoing transmission. Burkina Faso, Cameroon, Cote d'Ivoire, Sierra Leone, Tanzania, and Zambia have signed agreements with China to establish Institutional-based Networks of Cooperation between Africa and China on Malaria (INCAM). This communication platform will enable sustainable promotion of Africa–China cooperation to eliminate malaria.

## Conclusions

China’s efforts to eliminate malaria is impressive, and the country is dedicated to sharing its lessons learned in malaria elimination—including, but not limited to, the application of novel genetics-based approaches—with other nations through new initiatives, such as the Silk Road Economic Belt and the 21st Century Maritime Silk Road. In the coming years, China will promote international relationships and establish collaborative platforms on a wide range of topics in roughly 65 countries, including 20 African nations. China has implemented the Australia–China–Papua New Guinea Pilot Cooperation on Malaria Control Project, the UK–China–Tanzania Pilot Cooperation on Malaria Control Project, and BMGF-China-Tanzania Cooperative Project on Malaria Control.

## Data Availability

Not applicable.
